# The Impact of Chemotherapy and Body Mass Index on Cancer‐Related Fatigue in Colon Cancer Patients: A PROFILES‐Registry Study

**DOI:** 10.1002/cam4.70536

**Published:** 2025-01-06

**Authors:** Anneke Kastelein, Floortje Mols, Laura Kervezee, Niels H. Chavannes, Hans Gelderblom, Jacques Neefjes, Chris Hinnen

**Affiliations:** ^1^ Department of Cell and Chemical Biology, ONCODE Institute Leiden University Medical Center Leiden the Netherlands; ^2^ CoRPS ‐ Center of Research on Psychological Disorders and Somatic Diseases, Department of Medical and Clinical Psychology Tilburg University Tilburg the Netherlands; ^3^ Netherlands Comprehensive Cancer Organisation (IKNL) Utrecht the Netherlands; ^4^ Laboratory for Neurophysiology, Department of Cell and Chemical Biology Leiden University Medical Center Leiden the Netherlands; ^5^ Department of Public Health and Primary Care Leiden University Medical Center Leiden the Netherlands; ^6^ Department of Medical Oncology Leiden University Medical Center Leiden the Netherlands; ^7^ Department of Psycho‐Oncology LUMC Oncology Center Leiden the Netherlands

**Keywords:** BMI, cancer‐related fatigue, chemotherapy, colon cancer, quality of life

## Abstract

**Background:**

Inflammation has been reported to drive cancer‐related fatigue (CRF). As both obesity and chemotherapy promote inflammatory responses, obese cancer patients may be at risk of more severe CRF, especially when receiving chemotherapy.

**Methods:**

We analysed data of 333 colon cancer patients from four hospitals in the Netherlands (data derived from the PROCORE study). Fatigue was assessed with the general fatigue subscale of the Multidimensional Fatigue Inventory at four timepoints: at inclusion (T1), 4 weeks after surgery (T2), and 1 (T3) and 2 years (T4) after diagnosis. Linear mixed‐effects models were applied to evaluate the interaction between chemotherapy and body mass index (BMI) on the trajectory of fatigue.

**Results:**

The two‐way interactions between time and chemotherapy (*p* = 0.047) and between time and BMI on fatigue (*p* = 0.041) were significant. Patients scheduled for chemotherapy reported more fatigue during the treatment phase, while patients not treated with chemotherapy showed a stable trajectory. Obese patients reported more fatigue at follow‐up compared to patients with a healthy BMI. The three‐way interaction between time, chemotherapy and BMI was not significant (*p* = 0.39). However, obese chemotherapy‐treated patients reported the highest fatigue 2 years after treatment (12.8, 95% CI: 10.6–14.9). Their mean fatigue score was higher compared to baseline (9.2, 95% CI: 7.3–11.0, *p* < 0.001) and obese patients not treated with chemotherapy (9.6, 95% CI: 7.0–12.2, *p* = 0.02). Moreover, this group reported more fatigue compared to healthy (8.1, 95% CI: 5.5–10.9, *p* < 0.001) and overweight (9.7, 95% CI: 7.2–12.3, *p* = 0.019) chemotherapy‐treated patients.

**Conclusion:**

This study indicates that chemotherapy and BMI both influence long‐term fatigue in colon cancer patients. Proactive monitoring for CRF and lifestyle interventions may be needed for chemotherapy‐treated patients with a high BMI.

**Abbreviations**
AIC: Akaike information criteria
BMI: body mass index
CRF: cancer‐related fatigue
MFI: Multidimensional Fatigue Inventory
NCR: Netherlands Cancer Registry
PROFILES: Patient Reported Outcomes Following Initial treatment and Long term Evaluation of Survivorship

## 1 Introduction

Colon cancer is one of the most common cancers in men and women worldwide, and the incidence is increasing [1]. As life expectancy of colon cancer patients increases steadily following improved early detection and treatment [2], quality of life after cancer treatment becomes more relevant for patients and clinicians.

One of the most common and burdensome complaints during and after cancer treatment is cancer‐related fatigue (CRF) [3, 4, 5]. Patients with CRF experience a distressing, persistent, subjective sense of physical, emotional and/or cognitive tiredness or exhaustion which is not proportional to recent activity, is hardly relieved by resting, and interferes with usual functioning [6]. As a result, cancer survivors suffering from CRF may be unable to regain their pre‐cancer activity level and may face difficulties with daily life activities such as reading, getting out of bed, carrying out household chores, working or socialising [7]. CRF is most prominent during treatment but may persist for many years for a subset of colon cancer survivors [5].

While research efforts to understand CRF have increased in the past decades the pathophysiology of CRF remains unclear. A variety of potential biological mechanisms have been proposed, of which dysregulation of the immune system has been gaining much attention [8, 9]. Upregulation of pro‐inflammatory cytokines as a result of chemotherapy treatment has been suggested to lead to CRF via central nervous system signalling [8, 10]. Longitudinal studies in colorectal cancer patients have shown that patients receiving chemotherapy score higher on CRF [11], and long‐term CRF remains more common, compared to patients who did not receive chemotherapy [12]. Moreover, a recent study in breast cancer patients suggests that obese patients suffer more CRF compared to healthy‐weight patients before, during and after chemotherapy [13]. Since obesity also can promote upregulation of pro‐inflammatory cytokines and downregulation of anti‐inflammatory cytokines [14], obese cancer patients may be at risk of more severe and prolonged CRF, especially when receiving chemotherapy.

To our knowledge, the interaction between chemotherapy and obesity has not yet been investigated in patients with colon cancer. Therefore, the aim of these secondary analyses on the PROCORE study was to evaluate the interaction between chemotherapy and Body Mass Index (BMI) on the level and trajectory of fatigue over time in colon cancer patients following their diagnosis. Exploring the interactions between chemotherapy and BMI may provide opportunities for focused (preventive/lifestyle‐related) interventions to improve the quality of life of these cancer survivors.

## 2 Materials and Methods

### 2.1 Setting and Participants

This study is based on data derived from the PROCORE study. PROCORE was a prospective, population‐based study aimed at examining the longitudinal impact of colon and rectal cancer and its treatment on patient‐reported outcomes. Details of the data collection have been described previously [15]. In summary, the PROCORE study was performed by means of PROFILES, a registry for the physical and psychosocial impact of cancer and its treatment [16, 17]. PROFILES is linked to the Netherlands Cancer Registry (NCR), which collects clinical data from all newly diagnosed cancer patients in the Netherlands. Recruitment for PROCORE took place at four hospitals: Elisabeth‐TweeSteden Hospital, Catharina Hospital, Elkerliek Hospital and Máxima Medical Centre. All patients newly diagnosed with primary colorectal cancer between January 2016 and January 2019 were approached and, if eligible and informed consent was provided, included shortly after diagnosis (i.e., before start of treatment). Patients previously diagnosed with cancer (except basal skin cell carcinoma), those with cognitive impairment and those unable to read or write Dutch, were excluded. In practice, a limited number of patients who were previously diagnosed with cancer and those who already started treatment were included. For this secondary analysis, patients with rectal cancer were excluded, since rectal cancer is clinically and biologically different from colon cancer [18]. Moreover, treatment differs between these types of cancer: radiotherapy is commonly administered to patients with rectal cancer, while patients with colon cancer are rarely treated with radiotherapy [19]. Since radiotherapy is known to have a (short‐term) effect on CRF [20, 21] and the aim was to investigate the effect of chemotherapy, we excluded rectal cancer patients.

### 2.2 Data Collection

Eligible patients were invited by their research nurse or case manager and received an information package about the study. The package included an information letter, informed consent form and the first questionnaire (T1). Follow‐up questionnaires (online or on paper) were sent 4 weeks after surgery (T2), and 1 (T3) and 2 years (T4) after diagnosis. In case of non‐response, reminders were sent after 2 weeks. PROCORE was approved by the certified Medical Ethic Committee of Medical Research Ethics Committees United (registration number: NL51119.060.14).

### 2.3 Measures

In this analysis, participants were categorised based on their BMI at baseline: obese (≥ 30.0 kg/m^2^; *n* = 65), overweight (25.0–29.9 kg/m^2^; *n* = 159) and normal weight (18.0–24.9 kg/m^2^; *n* = 109). Participants with BMI < 18 kg/m^2^ were excluded from analysis. Height and weight (used to calculate BMI) were self‐reported by patients. Patients' sociodemographic (i.e., age, sex) and clinical (i.e., cancer type, clinical stage, treatment) information was available from the NCR. Education level was derived from the questionnaire.

### 2.4 Fatigue

Fatigue was assessed with the General Fatigue scale of the Multidimensional Fatigue Inventory (MFI). This 20‐item self‐report instrument was designed to measure fatigue and has been validated in cancer patients [22]. The questionnaire has an equal amount of positively and negatively worded items which are rated on a 5‐point Likert scale (e.g., “I feel tired”, “I feel rested”). It covers the five dimensions of fatigue: General Fatigue, Physical Fatigue, Mental Fatigue, Reduced Motivation and Reduced Activity. The General Fatigue subscale consists of 4 items, with the sum of scores ranging from 4 to 20. A higher score indicates a higher level of fatigue. For the MFI no absolute cut‐off point for fatigue exists.

### 2.5 Statistical Analyses

Baseline characteristics were determined. Linear mixed effects models with maximum likelihood estimation and an unstructured covariance matrix with a 2‐level structure (time‐level and patient‐level) were used. Mixed model analyses allow the number of observations per assessment to differ and therefore missing data were not imputed. A sequence of models was fitted to investigate fatigue from diagnosis until 2 years after diagnosis. Whether a more complex model fits the data better was determined based on the Akaike Information Criteria (AIC). A difference ≥ 4 between models was viewed as significant [23]. First, a model with no explanatory variables, only the intercept (i.e., an unconditional model), was fitted to the data to determine the extent of variance at the person and time level. Second, a model with only baseline characteristics (age, sex, education level) was calculated. Variables significantly associated with fatigue were used as covariates in the next models. Next, time as explanatory variable (unconditional growth model) was included to determine whether fatigue changed over time. Intercept and time were entered both as fixed and random effects as each subject may have its own unique intercept and slope. Third, the unconditional growth model was extended into a conditional growth model by including (i) possible covariates (i.e., age, sex, education level), (ii) chemotherapy (yes/no), (iii) baseline BMI and (iv) the interaction terms of time by chemotherapy, time by BMI and time by chemotherapy by BMI. By including the interaction terms, we were able to investigate whether fatigue develops differently over time depending on chemotherapy and BMI. Regression estimates and 95% confidence interval of fixed effects are presented. Analyses were done in SPSS version 23.

## 3 Results

In our analyses 333 eligible patients with colon cancer were included. Table 1 shows baseline clinical and demographic characteristics. Of all patients, 94 were treated with chemotherapy (28.2%). Relatively more males were included (60.0%). The majority of patients were overweight (47.7%) or obese (19.5%). Patients treated with and without chemotherapy were comparable with regard to age, BMI and education level. Between the different BMI categories patients were comparable with regards to age, education level and chemotherapy treatment. All patients completed baseline questionnaire, 292 (88%), 259 (78%) and 247 (74%) completed T2, T3 and T4, respectively.

**TABLE 1:** Demographics and disease characteristics of colon cancer patients at baseline.

	No. of patients (%)
Total no. of patients	333
Age (SD)	68.0 (9.14)
Sex	
Male	200 (60.0%)
Female	133 (39.9%)
Education level	
Low	35
Medium	211
High	82
Missing	5
Chemotherapy (yes)	94 (28.2%)
Pathological tumour stage	
I	108 (32.4%)
II	108 (32.4%)
III	109 (32.7%)
IV	7 (2.1%)
X	1 (0.3%)
BMI	
18–25	109 (32.7%)
25–30	159 (47.7%)
> 30	65 (19.5%)

*Note:* Education: low (no or primary school); medium (lower general secondary education or vocational training); high (pre‐university education, high vocational training, university).

Abbreviation: BMI, body mass index.

### 3.1 The Association Between Time and CRF

**FIGURE 1:**
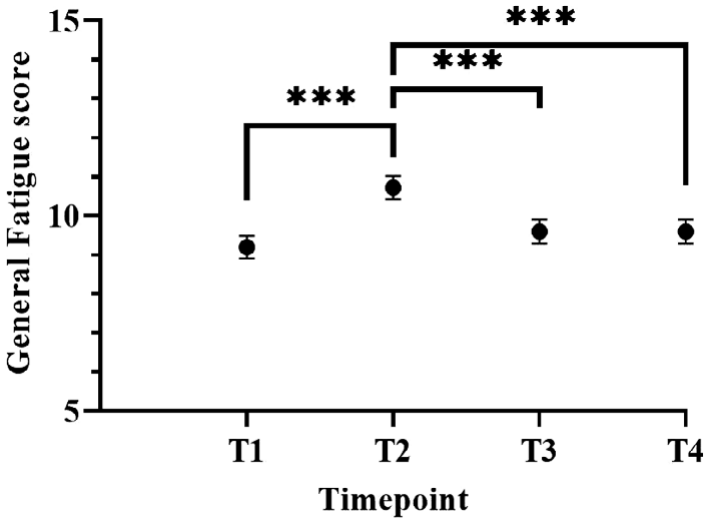
Trajectory of fatigue in colon cancer patients. Fatigue is significantly higher at T2 compared to other timepoints. Results are presented as estimated marginal mean scores accompanied with standard error of the mean intervals. Higher scores indicate more fatigue. Unconditional growth model with post hoc Bonferroni multiple comparison. T1: At diagnosis, T2: 4 weeks after surgery. T3: 1 year post diagnosis. T4: 2 years after diagnosis. ***p < 0.001.

Intraclass correlation coefficients of the unconditional model showed that 56% of the variance was at the person level and the remaining 43% was at the time level. These results indicate that scores on CRF differed sufficiently between patients and over time to justify a two‐level model. Next, we entered possible covariates (age, sex, education level) into the model. Only sex was found to be significantly different (*p* = 0.0035) indicating that women scored higher (10.1, 95% CI 9.4–10.8) on fatigue than men (9.0, 95% CI 8.4–9.7). Next, we computed an unconditional growth model by entering time as a fixed effect into the model. This model fitted the data better than the unconditional model and showed that time impacted the level of fatigue (*p* < 0.001). In this model, fatigue significantly increased from pre‐treatment (9.2, 95% CI 8.6–9.6) to post‐surgery (10.4, 95%CI 10.1–11.3) and decreased during follow‐up at 1 (9.59, 95% CI 9.0–10.2) and 2 years (9.6, 95% CI 8.9–10.2; Figure 1, Table S1).

Moreover, the conditional growth model with time, sex, chemotherapy, BMI and the interaction terms showed that this model fitted the data as good as the unconditional growth model. A significant effect of time (*p* < 0.001), sex (*p* = 0.003), BMI (*p* = 0.009) and the interaction between time and chemotherapy (*p* = 0.047), and between time and BMI (*p* = 0.041) on fatigue levels was found. No significant three‐way interaction between time, chemotherapy and BMI was found (*p* = 0.39).

### 3.2 The Interaction Between Time and Chemotherapy

**FIGURE 2:**
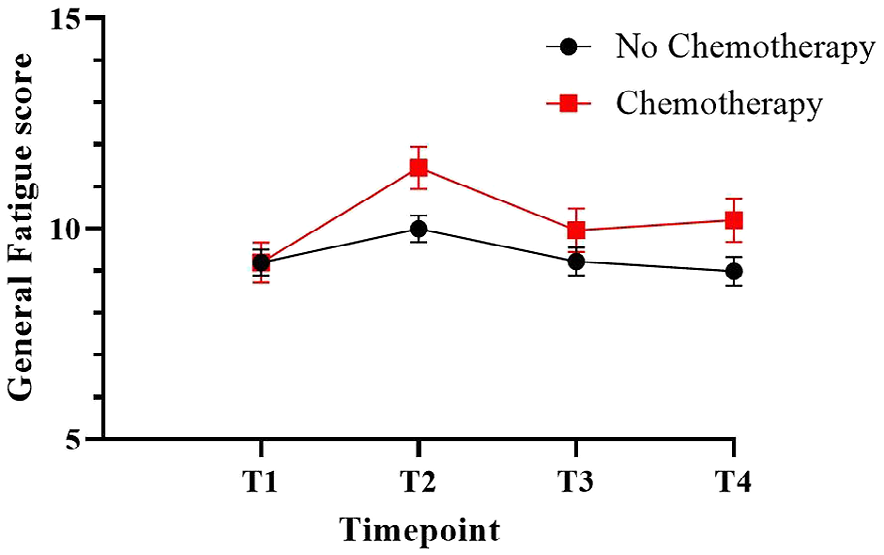
The fatigue trajectory differs between treatment groups. In the group that receives chemotherapy, fatigue increase at T2 compared to baseline. In the non‐chemotherapy group, fatigue does not change significantly over time. Results are presented as estimated marginal mean scores and accompanied with standard error of the mean intervals. Higher scores indicate more fatigue. T1: At diagnosis, T2: 4 weeks after surgery. T3: 1 year post diagnosis. T4: 2 years after diagnosis.

Chemotherapy‐treated patients reported more fatigue during treatment phase (11.5, 95%CI 10.5–12.4) compared to pre‐treatment (9.2, 95%CI 8.3–10.1), 1 year (10.0, 95%CI 9.0–11.0) and 2 year (10.2, 95%CI 9.2–11.2) follow‐up (Figure 2 and Table S2). No significant differences over time are seen in the group that did not receive chemotherapy.

### 3.3 The Interaction Between BMI and Chemotherapy

**FIGURE 3:**
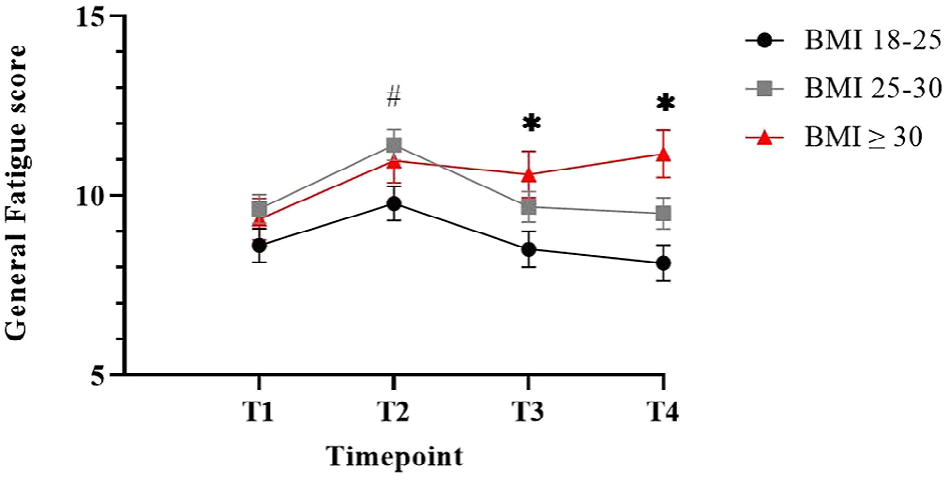
Fatigue over time in different BMI groups. Patients with a BMI ≥ 30 reported more fatigue 2 years after diagnosis compared to patients with a healthy BMI. Results are presented as adjusted mean scores accompanied with standard error of the mean intervals. Higher scores indicate more fatigue. T1: At diagnosis, T2: 4 weeks after surgery. T3: 1 year post diagnosis. T4: 2 years after diagnosis. *Difference between BMI ≥ 30 and BMI 18–25 *p* < 0,05; # difference between BMI 25–30 and BMI 18–25 *p* < 0.05.

The significant interaction between time and BMI shows that patients with a higher BMI reported a different fatigue trajectory compared to patients with a lower BMI (Figure 3, Table S3). Post hoc pairwise comparisons revealed that while no differences in fatigue are present between the BMI groups at baseline, differences arise during and after treatment. Overweight patients reported more fatigue during treatment phase compared to healthy weight patients (mean difference 1.6, *p* = 0.04). During follow‐up, scores of overweight patients decrease again, while obese patients reported more fatigue 1 year (mean difference 2.2, *p* = 0.04) and 2 years after diagnosis (mean difference 2.6, *p* = 0.01) compared to patients with a healthy BMI.

### 3.4 Chemotherapy, BMI and the Trajectory of Fatigue

**FIGURE 4:**
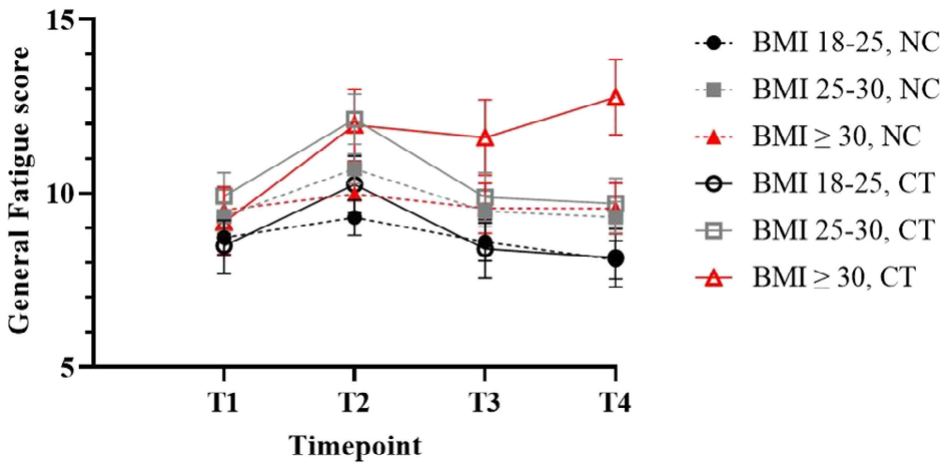
Chemotherapy‐treated obese patients remain fatigued over time. Patients with a BMI ≥ 30 treated with chemotherapy reported more fatigue during treatment phase (T2) compared to baseline. These scores remained higher up to 2 years after diagnosis compared to chemotherapy patients with a lower BMI and patients that did not receive chemotherapy. Results are presented as estimated marginal mean scores accompanied with standard error of the mean intervals. Higher scores indicate more fatigue. T1: At diagnosis, T2: 4 weeks after surgery. T3: 1 year post diagnosis. T4: 2 years after diagnosis, CT, chemotherapy treated, NC, no chemotherapy treatment.

The three‐way interaction between time, chemotherapy and BMI was not significant. However, post hoc analyses showed that the level of CRF at a specific assessment point does depend on BMI and chemotherapy. See Table 2 for an overview of the estimated effect of the covariates and interactions on the mean fatigue score. Chemotherapy‐treated patients with a BMI ≥ 30 reported the highest levels of fatigue specifically at 2 years after diagnosis (Figure 4 and Table S4).

**TABLE 2:** Estimated effect of the covariates and interactions on the mean fatigue score relative to the reference group.

	Estimate	95% CI	*p*
Intercept^a^	13.41	11.2, 15.6	0.000
T1	−3.59	−5.6, −1.6	0.000
T2	−0.80	−2.9, 1.3	0.456
T3	−1.16	−3.3, 1	0.296
Sex	−1.29	−2.1, −0.5	0.003
Chemotherapy	−3.20	−5.8, −0.6	0.015
BMI 18–25	−4.62	−7.3, −1.9	0.001
BMI 25–30	−3.06	−5.6, −0.5	0.019
T1 * Chemotherapy	3.55	1.1, 6	0.004
T2 * chemotherapy	1.21	−1.3, 3.7	0.344
T3* chemotherapy	1.17	−1.4, 3.8	0.378
T1 * BMI 18–25	3.94	1.4, 6.5	0.002
T1 * BMI 25–30	3.81	1.4, 6.2	0.002
T2 * BMI 18–25	2.91	0.3, 5.5	0.029
T2 * BMI 25–30	3.22	0.7, 5.7	0.012
T3 * BMI 18–25	1.42	−1.3, 4.1	0.301
T3 * BMI 25–30	1.33	−1.2, 3.9	0.307
T1 * Chemotherapy * BMI 18–25	−0.12	−3.1, 2.8	0.937
T1 * Chemotherapy * BMI 25–30	−0.93	−3.7, 1.8	0.505
T2 * Chemotherapy * BMI 18–25	1.03	−2.1, 4.1	0.512
T2 * Chemotherapy * BMI 25–30	0.56	−2.4, 3.5	0.706
T3 * Chemotherapy * BMI 18–25	2.24	−1, 5.5	0.174
T3 * Chemotherapy * BMI 25–30	1.63	−1.4, 4.7	0.290
T4 * Chemotherapy * BMI 18–25	3.15	−0.1, 6.4	0.057
T4 * Chemotherapy * BMI 25–30	2.80	−0.3, 5.9	0.073

*Note:* Post hoc results from the final conditional growth model by including sex, time, chemotherapy, baseline BMI, and the interaction terms of time by chemotherapy by BMI. The values in the table represent the estimated effect of the covariates and interactions on the mean fatigue score relative to the reference group: patients with a BMI ≥ 30 who received chemotherapy at T4. T1: at diagnosis, T2: 4 weeks after surgery, T3: 1 year post diagnosis, T4: 2 years after diagnosis.

Abbreviation: BMI, body mass index.

^a^ BMI > 30 who received chemotherapy with mean fatigue score of 12.76 at T4.

These patients reported a mean score of 12.8 (95%CI: 10.6–14.9) at T4, 3.6 point higher compared to baseline T1 (9.2, 95%CI: 7.3–11.0), *p* < 0.001. Patients with a BMI of 18–25 and 25–30 who received chemotherapy reported a significantly lower mean fatigue score of 8.1 (95%CI: 5.5–10.9, *p* < 0.001) and 9.7 (95%CI:7.2–12.3, *p* = 0.019), respectively, at T4. Moreover, chemotherapy‐treated patients with a BMI ≥ 30 reported significantly higher levels of fatigue at T4 than their non‐chemotherapy treated counterparts (9.6, 95%CI: 7.0–12.2, *p* = 0.015).

## 4 Discussion

CRF is a serious long‐term effect of cancer treatment that dramatically decreases the quality of life of cancer survivors [6]. Previous research suggests that a high BMI and chemotherapy may interact to exacerbate CRF, but to what extent this applies to colon cancer patients remained unclear.

When analysing colon cancer patients as one group, fatigue increased during treatment phase, but recovered to pre‐treatment levels during follow‐up. Yet, when assessing the effects of chemotherapy and BMI, our study reveals several interesting observations. First, as expected, general fatigue was higher during treatment phase for chemotherapy‐treated patients. As T2 was sent out 4 weeks after surgery, it is likely that not all patients scheduled for chemotherapy had started treatment when answering the questionnaire, potentially underrepresenting the effect of chemotherapy. Nonetheless, the interaction between time and chemotherapy showed that chemotherapy‐treated patients were more fatigued during the treatment phase, while non‐chemotherapy‐treated patients showed a stable pattern. This is in line with existing literature in colorectal cancer patients [11, 12]. Second, overweight patients reported more fatigue during treatment phase compared to healthy weight patients. Obese patients remained more fatigued during follow‐up compared to patients with a healthy BMI. This corroborates with earlier reported studies in colorectal cancer survivors that reported an association between higher BMI and fatigue [11, 24]. Thirdly, obese chemotherapy‐treated patients did not recover to pre‐treatment levels but rather continued to feel fatigued. Two years after treatment, this group of patients reported more fatigue compared to overweight (3.1 points higher) and healthy weight patients (4.6 points higher) as well as patients who did not receive chemotherapy at all (3.2 points higher). Given that a two‐point difference on the MFI subscales is considered clinically meaningful, the observed long‐term differences are substantial and of significant clinical relevance. These differences are highly pertinent for both patients and clinicians, as they may reflect considerable changes in functional status of cancer survivors [25]. The results are comparable with a recent study among 565 women with breast cancer treated with chemotherapy, reporting that obese patients (BMI ≥ 30) suffer more CRF compared to normal weight patients before, during and after chemotherapy [13]. Interestingly, BMI did not affect fatigue prior to treatment in our study. Differences may arise from differences in the time of measurement. In the breast cancer study, patients were assessed 7 days prior to chemotherapy and may have already been exposed to surgery and radiotherapy [13], while in our study patients had not received any treatment prior to the first measurement (T1). A high BMI itself does not appear to have a significant effect on MFI scores in healthy women [26]. The data in our study implies that the effect of BMI on fatigue is exacerbated when the patients are treated with chemotherapy, especially in the long term.

How the interplay of obesity and chemotherapy observed in this study results in an exacerbation of CRF in colon cancer patients remains to be clarified. Several lines of research have confirmed that obesity leads to chronic low‐grade inflammation of the adipose tissue [27]. Weight gain and obesity cause a phenotypic switch of the adipose tissue, resulting in the release of proinflammatory cytokines [27]. Pro‐inflammatory cytokines act on the central nervous system through several routes [28] and can elicit local inflammation resulting in altered brain functioning [29]. Conditions of chronic inflammation exacerbate sickness‐like behaviours, characterised by poor sleep, reduced food intake, fatigue and pain complaints, social withdrawal, anhedonia, and memory and learning difficulties [28] that develop in response to acute peripheral inflammation, symptoms that are often seen with CRF. This phenomenon has been shown in several animal models with inflammatory conditions, and might be due to an effect called ‘priming’: exposure to a priming stimulus leads to an exaggerated production of pro‐inflammatory cytokines when exposed to a triggering stimulus [28]. Preclinical experiments have shown that obese animals show a prolonged and more pronounced behavioural response when exposed to an inflammatory challenge [30, 31]. Chemotherapy might pose such an inflammatory challenge—alteration of cytokine profiles have been reported after standard doses of chemotherapy regimens [3, 32]. Disruption of the balance between pro– and anti‐inflammatory cytokines following chemotherapy treatment may lead to a more intensified inflammatory response in obese cancer patients and result in chronic CRF. One may speculate that this may further be accelerated as obese patients may accumulate more drugs in fat tissues, resulting in a different release and altered inflammatory responses. Importantly, the effect of inflammation on brain functioning may be chronic, even if peripheral inflammation is not detectable anymore [33]. For this study, we analysed previously collected data from the PROCORE study. No data was currently available to identify biological pathways. Other mechanisms may play a role, such as alteration of gut microbiome, circadian rhythm disruption or HPA‐axis dysregulation following chemotherapy [9]. Future (pre)clinical studies might examine the mechanisms of (neuro) inflammatory changes following chemotherapy treatment as well as the persistence of these changes by collecting biomarkers and proactive monitoring. Future research may also focus on lifestyle interventions to avoid fatigue exacerbation.

The value of this study includes the homogeneous sample of colon cancer patients as well as longitudinal design. The study has some limitations. No data was available on the chemotherapy type or dosing schedule. Higher body weight likely resulted in higher absolute dosing, as patients are dosed according to body surface area. Fatigue may therefore result from exposure to a higher dose of chemotherapy which we have not been able to account for. Moreover, weight and height were self‐reported and may be subject to response bias. In addition, it is not known why patients were lost to follow‐up, as such the effect of it on our analysis remains unclear. Furthermore, a very small number of patients received chemotherapy prior to the baseline questionnaire, which could have impacted the strength of our findings. Finally, the present results may not be generalisable to a broader group of (gastrointestinal) cancer patients. Due to the relatively low number of stage IV patients who participated in the study, our findings should be extrapolated to this group with care. Nevertheless, this study shows that further investigation of the interaction between chemotherapy, BMI and CRF is indispensable.

## 5 Conclusion

Our study indicates that colon cancer patients with a BMI ≥ 30 who receive chemotherapy treatment have a higher risk of remaining fatigued over longer periods, in contrast to patients that do not receive chemotherapy treatment or are not obese. Regular monitoring for CRF in this group as well as proactive communication with these patients on the possibility that their BMI may impact chronic fatigue may be needed. Moreover, lifestyle modifying coaching, including dietary and exercise interventions, may be beneficial prior, during or after treatment.

## 6 Précis

Results from this study indicate that colon cancer patients with a BMI≥ 30 develop chronic fatigue after treatment with chemotherapy. Two years after treatment, these patients reported significantly higher fatigue levels compared to patients with a lower BMI or patients who did not receive chemotherapy at all.

## Author Contributions

**Anneke Kastelein:** conceptualisation, methodology, writing – original draft, visualisation. **Floortje Mols:** methodology, investigation, resources, writing – review and editing. **Laura Kervezee:** validation, writing – review and editing. **Niels H. Chavannes:** supervision, writing – review and editing. **Hans Gelderblom:** supervision, writing – review and editing. **Jacques Neefjes:** supervision, writing – review and editing. **Chris Hinnen:** conceptualisation, methodology, formal analysis, writing – original draft.

## Ethics Statement

The PROCORE study was approved by the certified Medical Ethic Committee of Medical Research Ethics Committees United (registration number: NL51119.060.14).

## Conflicts of Interest

The authors declare no conflicts of interest.

## Supporting information


Data S1.

## Data Availability

The data is freely available for non‐commercial scientific research, subject to study question, privacy and confidentiality restrictions, and registration (www.profilesregistry.nl).

## References

[1] H. Sung , J. Ferlay , R. L. Siegel , et al., “Global Cancer Statistics 2020: GLOBOCAN Estimates of Incidence and Mortality Worldwide for 36 Cancers in 185 Countries,” CA: A Cancer Journal for Clinicians 71, no. 3 (2021): 209–249, 10.3322/CAAC.21660. 33538338

[2] Netherlands Comprehensive Cancer Organisation (IKNL) , Netherlands Cancer Registery (NCR), https://www.iknl.nl/en/ncr/ncr‐data‐figures.

[3] X. S. Wang , L. A. Williams , S. Krishnan , et al., “Serum sTNF‐R1, IL‐6, and the Development of Fatigue in Patients With Gastrointestinal Cancer Undergoing Chemoradiation Therapy,” Brain, Behavior, and Immunity 26, no. 5 (2012): 699–705, 10.1016/J.BBI.2011.12.007. 22251605 PMC3355215

[4] C. J. Han , G. S. Yang , and K. Syrjala , “Symptom Experiences in Colorectal Cancer Survivors After Cancer Treatments: A Systematic Review and Meta‐Analysis,” Cancer Nursing 43, no. 3 (2020): E132–E158, 10.1097/NCC.0000000000000785. 32000174 PMC7182500

[5] M. S. Y. Thong , F. Mols , X. S. Wang , V. E. P. P. Lemmens , T. J. Smilde , and L. V. Van De Poll‐Franse , “Quantifying Fatigue in (Long‐Term) Colorectal Cancer Survivors: A Study From the Population‐Based Patient Reported Outcomes Following Initial Treatment and Long Term Evaluation of Survivorship Registry,” European Journal of Cancer 49, no. 8 (2013): 1957–1966, 10.1016/j.ejca.2013.01.012. 23453750 PMC3676930

[6] A. M. Berger , K. Mooney , A. Alvarez‐Perez , et al., “Cancer‐Related Fatigue, Version 2.2015,” Journal of the National Comprehensive Cancer Network 13, no. 8 (2015): 1012–1039, 10.6004/JNCCN.2015.0122. 26285247 PMC5499710

[7] T. I. Bootsma , M. P. J. J. Schellekens , R. A. M. M. van Woezik , M. L. van der Lee , and J. Slatman , “Experiencing and Responding to Chronic Cancer‐Related Fatigue: A Meta‐Ethnography of Qualitative Research,” Psycho‐Oncology 29, no. 2 (2020): 241–250, 10.1002/pon.5213. 31442340 PMC7027742

[8] J. E. Bower , “Cancer‐Related Fatigue—Mechanisms, Risk Factors, and Treatments,” Nature Reviews. Clinical Oncology 11, no. 10 (2014): 597–609, 10.1038/nrclinonc.2014.127. PMC4664449 25113839

[9] D. García‐González , J. Medino‐Muñoz , M. Romero‐Elías , J. García‐Foncillas , and A. Ruiz‐Casado , “Biological Mechanisms of Cancer‐Related Fatigue in Breast Cancer Survivors After Treatment: A Scoping Review,” Journal of Cancer Survivorship 6 (2023): 1–31, 10.1007/S11764-023-01477-Z/TABLES/1. 37930591

[10] A. Jager , S. Sleijfer , and C. C. D. van der Rijt , “The Pathogenesis of Cancer Related Fatigue: Could Increased Activity of Pro‐Inflammatory Cytokines Be the Common Denominator?,” European Journal of Cancer 44, no. 2 (2008): 175–181, 10.1016/j.ejca.2007.11.023. 18162394

[11] O. Husson , F. Mols , L. V. van de Poll‐Franse , and M. S. Y. Thong , “The Course of Fatigue and Its Correlates in Colorectal Cancer Survivors: A Prospective Cohort Study of the PROFILES Registry,” Supportive Care in Cancer 23, no. 11 (2015): 3361–3371, 10.1007/S00520-015-2802-X. 26123601 PMC4584107

[12] J. L. Vardy , H. M. Dhillon , G. R. Pond , et al., “Fatigue in People With Localized Colorectal Cancer Who Do and Do Not Receive Chemotherapy: A Longitudinal Prospective Study,” Annals of Oncology 27, no. 9 (2016): 1761–1767, 10.1093/annonc/mdw252. 27443634 PMC4999562

[13] J. E. Inglis , M. C. Janelsins , E. Culakova , et al., “Longitudinal Assessment of the Impact of Higher Body Mass Index on Cancer‐Related Fatigue in Patients With Breast Cancer Receiving Chemotherapy,” Supportive Care in Cancer 28, no. 3 (2020): 1411–1418, 10.1007/S00520-019-04953-4/FIGURES/2. 31267279 PMC7243469

[14] J. J. Fuster , N. Ouchi , N. Gokce , and K. Walsh , “Obesity‐Induced Changes in Adipose Tissue Microenvironment and Their Impact on Cardiovascular Disease,” Circulation Research 118, no. 11 (2016): 1786–1807, 10.1161/CIRCRESAHA.115.306885. 27230642 PMC4887147

[15] C. S. Bonhof , L. V. van de Poll‐Franse , D. K. Wasowicz , et al., “The Course of Peripheral Neuropathy and Its Association With Health‐Related Quality of Life Among Colorectal Cancer Patients,” Journal of Cancer Survivorship 15 (2021): 190–200, 10.1007/s11764-020-00923-6. 33185839 PMC7966630

[16] L. V. Van De Poll‐Franse , N. Horevoorts , D. Schoormans , et al., “Measuring Clinical, Biological, and Behavioral Variables to Elucidate Trajectories of Patient‐Reported Outcomes: The PROFILES Registry,” Journal of the National Cancer Institute 114, no. 6 (2022): 800–807, 10.1093/jnci/djac047. 35201353 PMC9194631

[17] L. V. Van De Poll‐Franse , N. Horevoorts , E. M. Van , et al., “The Patient Reported Outcomes Following Initial Treatment and Long Term Evaluation of Survivorship Registry: Scope, Rationale and Design of an Infrastructure for the Study of Physical and Psychosocial Outcomes in Cancer Survivorship Cohorts,” European Journal of Cancer 47, no. 14 (2011): 2188–2194, 10.1016/J.EJCA.2011.04.034. 21621408

[18] K. Tamas , A. M. E. Walenkamp , E. G. E. de Vries , et al., “Rectal and Colon Cancer: Not Just a Different Anatomic Site,” Cancer Treatment Reviews 41, no. 8 (2015): 671–679, 10.1016/J.CTRV.2015.06.007. 26145760

[19] Dutch Federation of Medical Specialists , Dutch Clinical Practice Guidelines accessed November 25, 2022, https://richtlijnendatabase.nl/.

[20] C. L. Barker , J. A. Routledge , D. J. Farnell , R. Swindell , and S. E. Davidson , “The Impact of Radiotherapy Late Effects on Quality of Life in Gynaecological Cancer Patients,” British Journal of Cancer 100, no. 10 (2009): 1558–1565, 10.1038/sj.bjc.6605050. 19384297 PMC2696756

[21] B. Yucel , E. A. Akkaş , Y. Okur , et al., “The Impact of Radiotherapy on Quality of Life for Cancer Patients: A Longitudinal Study,” Supportive Care in Cancer 22, no. 9 (2014): 2479–2487, 10.1007/S00520-014-2235-Y/TABLES/5. 24728584

[22] E. M. A. Smets , B. Garssen , B. Bonke , and J. C. J. M. De Haes , “The Multidimensional Fatigue Inventory (MFI) Psychometric Qualities of an Instrument to Assess Fatigue,” Journal of Psychosomatic Research 39, no. 3 (1995): 315–325, 10.1016/0022-3999(94)00125-O. 7636775

[23] K. P. Burnham and D. R. Anderson , “Multimodel Inference: Understanding AIC and BIC in Model Selection,” Sociological Methods & Research 33, no. 2 (2004): 261–304, 10.1177/0049124104268644.

[24] P. A. J. Vissers , R. B. Martucci , F. Mols , et al., “The Impact of Body Mass Index and Waist Circumference on Health‐Related Quality of Life Among Colorectal Cancer Survivors: Results from the PROFILES Registry,” Nutrition and Cancer 69, no. 8 (2017): 1177–1184, 10.1080/01635581.2017.1367938. 29035593

[25] A. Purcell , J. Fleming , S. Bennett , B. Burmeister , and T. Haines , “Determining the Minimal Clinically Important Difference Criteria for the Multidimensional Fatigue Inventory in a Radiotherapy Population,” Supportive Care in Cancer 18, no. 3 (2010): 307–315, 10.1007/S00520-009-0653-Z/TABLES/3. 19468758

[26] M. C. Rodriguez , M.‐y. J. El , A. Casas‐barrag , F. Molina , and B. Rueda‐medina , “Composition With Pain, Disease Activity, Fatigue, Sleep and Anxiety in Women With Fibromyalgia,” Nutrients 11, no. 5 (2019): 13, https://www.ncbi.nlm.nih.gov/pubmed/31137906. 10.3390/nu11051193 PMC6566359 31137906

[27] T. Kawai , M. V. Autieri , and R. Scalia , “Inflammation: From Cellular Mechanisms to Immune Cell Education: Adipose Tissue Inflammation and Metabolic Dysfunction in Obesity,” American Journal of Physiology. Cell Physiology 320, no. 3 (2021): C375–C391, 10.1152/AJPCELL.00379.2020. 33356944 PMC8294624

[28] R. Dantzer , J. C. O'Connor , G. G. Freund , R. W. Johnson , and K. W. Kelley , “From Inflammation to Sickness and Depression: When the Immune System Subjugates the Brain,” Nature Reviews Neuroscience 9, no. 1 (2008): 46–56, 10.1038/nrn2297. 18073775 PMC2919277

[29] B.‐N. Lee , R. Dantzer , K. E. Langley , et al., “A Cytokine‐Based Neuroimmunologic Mechanism of Cancer‐Related Symptoms,” Neuroimmunomodulation 11 (2004): 279–292, 10.1159/000079408. 15316238

[30] C. B. Lawrence , D. Brough , and E. M. Knight , “Obese Mice Exhibit an Altered Behavioural and Inflammatory Response to Lipopolysaccharide,” Disease Models & Mechanisms 5, no. 5 (2012): 649–659, 10.1242/DMM.009068. 22328591 PMC3424462

[31] J. Pohl , M. Sheppard , G. N. Luheshi , and B. Woodside , “Diet‐Induced Weight Gain Produces a Graded Increase in Behavioral Responses to an Acute Immune Challenge,” Brain, Behavior, and Immunity 35 (2014): 43–50, 10.1016/j.bbi.2013.09.002. 24026015

[32] T. Eyob , T. Ng , R. Chan , and A. Chan , “Impact of Chemotherapy on Cancer‐Related Fatigue and Cytokines in 1312 Patients: A Systematic Review of Quantitative Studies,” Current Opinion in Supportive and Palliative Care 10, no. 2 (2016): 165–179, 10.1097/SPC.0000000000000205. 27043288

[33] J. E. Inglis , A. S. Kleckner , P. J. Lin , et al., “Excess Body Weight and Cancer‐Related Fatigue, Systemic Inflammation, and Serum Lipids in Breast Cancer Survivors,” Nutrition and Cancer 73, no. 9 (2021): 1676–1686, 10.1080/01635581.2020.1807574. 32812824 PMC7904100

