# Supplementary material for: The Impact of Chemotherapy and Body Mass Index on Cancer‐Related Fatigue in Colon Cancer Patients: A PROFILES‐Registry Study
Source: Cancer Med. 2025 Jan 6;14(1):e70536. doi: 10.1002/cam4.70536 (PMC11702450; doi:10.1002/cam4.70536)
Supplement: Supplementary file 1 — Data S1. [file CAM4-14-e70536-s001.docx]

**Supplemental**

**Supplemental Table 1.** Model predicted means for general fatigue per timepoint in colon cancer patients.

| Timepoint | Mean Score | SE |
| --- | --- | --- |
| T1 | 9.19 | 0.29 |
| T2 | 10.72 | 0.30 |
| T3 | 9.59 | a.31 |
| T4 | 9.60 | 0.31 |

Results follow from unconditional growth model with time as fixed effect. SE: standard error.

**Supplemental Table 2.** Model predicted means for general fatigue per timepoint between chemotherapy and non-chemotherapy treated colon cancer patients.

|  | **No Chemotherapy** | | **Chemotherapy** | |
| --- | --- | --- | --- | --- |
| Timepoint | Mean Score | SE | Mean score | SE |
| T1 | 9.20 | 0.31 | 9.19 | 0.48 |
| T2 | 10.00 | 0.32 | 11.45 | 0.51 |
| T3 | 9.22 | 0.34 | 9.97 | 0.52 |
| T4 | 8.99 | 0.34 | 10.20 | 0.52 |

Model predicted estimated marginal mean scores per timepoint. Scores follow from the conditional growth model with time, sex, chemotherapy and the interaction terms between time and chemotherapy. SE: Standard error.

**Supplemental Table 3.** Model predicted means for general fatigue per timepoint between different BMI categories.

|  | **BMI 18-25** | | **BMI 25-30** | | **BMI > 30** | |
| --- | --- | --- | --- | --- | --- | --- |
| Timepoint | Mean Score | SE | Mean Score | SE | Mean Score | SE |
| T1 | 8.61 | 0.47 | 9.63 | 0.40 | 9.35 | 0.58 |
| T2 | 9.78 | 0.48 | 11.42 | 0.42 | 10.97 | 0.62 |
| T3 | 8.51 | 0.50 | 9.69 | 0.43 | 10.59 | 0.65 |
| T4 | 8.12 | 0.50 | 9.51 | 0.43 | 11.16 | 0.66 |

Model predicted estimated marginal mean scores per timepoint. Scores follow from the conditional growth model with time, sex, BMI and the interaction terms between time and BMI. SE: Standard error.

**Supplemental Table 4.** Model predicted means for general fatigue per timepoint between patients from different BMI categories, treated with or without chemotherapy.

|  | **BMI 18-25, NC** | | **BMI 25-30, NC** | | **BMI > 30, NC** | | **BMI 18-25, CT** | | **BMI 25-30, CT** | | **BMI > 30, CT** | |
| --- | --- | --- | --- | --- | --- | --- | --- | --- | --- | --- | --- | --- |
| Timepoint | Mean Score | SE | Mean Score | SE | Mean Score | SE | Mean Score | SE | Mean Score | SE | Mean Score | SE |
| T1 | 8.73 | 0.49 | 9.34 | 0.41 | 9.52 | 0.66 | 8.49 | 0.81 | 9.92 | 0.68 | 9.17 | 0.95 |
| T2 | 9.30 | 0.51 | 10.70 | 0.43 | 9.98 | 0.69 | 10.25 | 0.82 | 12.13 | 0.72 | 11.97 | 1.04 |
| T3 | 8.61 | 0.54 | 9.49 | 0.44 | 9.58 | 0.72 | 8.40 | 0.84 | 9.88 | 0.72 | 11.61 | 1.09 |
| T4 | 8.09 | 0.55 | 9.31 | 0.45 | 9.57 | 0.74 | 8.14 | 0.83 | 9.71 | 0.73 | 12.76 | 1.09 |

Model predicted estimated marginal mean scores per timepoint. Scores follow from the final conditional growth model by including sex, time, chemotherapy, BMI, and the interaction terms of time by chemotherapy by BMI. NC: Not treated with chemotherapy, CT: Treated with chemotherapy, SE: Standard error
